# Pharmacokinetics and Bioequivalence of a Novel Extended‐Release Formulation of Methylphenidate Hydrochloride for Attention‐Deficit/Hyperactivity Disorder

**DOI:** 10.1002/cpdd.1577

**Published:** 2025-08-12

**Authors:** Ann C. Childress, Ahmad AL‐Sabbagh, Jeffrey H. Newcorn

**Affiliations:** ^1^ Center for Psychiatry and Behavioral Medicine Las Vegas NV USA; ^2^ Alora Pharmaceuticals, LLC Alpharetta GA USA; ^3^ Department of Psychiatry Icahn School of Medicine at Mount Sinai New York NY USA

**Keywords:** ADHD, attention‐deficit/hyperactivity disorder, extended‐release, methylphenidate, pharmacokinetics

## Abstract

Extended‐release (ER) formulations of the stimulant methylphenidate are commonly used to treat attention‐deficit/hyperactivity disorder in both children and adults. Previous studies have shown that the clinical effectiveness of long‐acting methylphenidate formulations is closely tied to the drug's pharmacokinetic (PK) profile, highlighting the need for consistency in drug exposure. ODX‐methylphenidate ER uses an osmotic pump design to provide controlled release of drug over the course of the day. In these similarly designed 4‐period replicate crossover studies, the PK profile of ODX‐methylphenidate ER was compared to the reference product, osmotic release oral system (OROS)‐methylphenidate ER, in healthy subjects in the fasted state. In the first trial (N = 60), a single 72‐mg tablet of ODX‐methylphenidate ER was compared to two 36‐mg tablets of OROS‐methylphenidate ER, while in the second trial (N = 36), a single 54‐mg tablet of ODX‐methylphenidate ER was compared to a 54‐mg tablet of OROS‐methylphenidate ER. The 2 studies had very comparable results, demonstrating similar PK parameters for the 2 products, including during the critical 7‐12‐hour postdose window. Statistical bioequivalence between the 2 formulations was confirmed for maximum drug concentration, area under the concentration‐time curve from time 0 to 3 hours after dosing (AUC_0‐3 h_), AUC from 3 to 7 hours after dosing, AUC from 7 to 12 hours after dosing, and AUC from time 0 extrapolated to infinity in both trials. Safety and tolerability were similar for both products and in both trials, with no serious adverse events reported.

Attention‐deficit/hyperactivity disorder (ADHD) is a highly prevalent neurodevelopmental disorder characterized by inattention, hyperactivity, and impulsivity.^1^ Approximately 10% of children and adolescents in the United States have been diagnosed with ADHD, and adult prevalence has been estimated to be around 3%.^2^, ^3^ The functional impairments related to ADHD can have significant negative impacts on quality of life for those with ADHD and their families, so symptom management is of critical importance.^1^

The stimulants methylphenidate and amphetamine are recommended as first‐line treatments for ADHD symptom management for children, adolescents, and adults.^4^ Amphetamine functions to increase the availability of dopamine and norepinephrine at the synaptic cleft, and once‐daily extended‐release (ER) formulations have been shown to improve the hyperactivity, impulsivity, and inattention associated with ADHD, as well as health‐related quality of life.^5^ Methylphenidate has a similar mechanism of action in increasing the synaptic availability of both dopamine and norepinephrine. Although there is no conclusive evidence supporting the use of 1 drug class over the other, some studies show a particularly favorable balance of response and tolerability for methylphenidate in children and adolescents.^4^, ^6^

Various long‐acting forms of both drug classes have been developed to provide full waking day coverage and reduce the burden of the multiple daily doses usually required when using immediate‐release formulations.^7^ Several different drug delivery methods have been used to prolong methylphenidate delivery over the day, including osmotic delivery systems, various types of bead technology, pH‐dependent polymer coatings, and ion‐exchange resins.^4^ A delayed‐release/ER option for evening dosing is also available, as are transdermal patches that may be useful in those with gastrointestinal issues and those with difficulty swallowing tablets, including children.^4^, ^8^

The pharmacokinetic (PK) profiles of methylphenidate formulations are closely tied to their therapeutic response profile over the day.^7^ Different drug delivery systems can significantly affect PK profiles of the drug, which can lead to important differences in efficacy over the course of the day.^4^ For instance, some formulations produce a PK curve with a single peak, while others, particularly those using bead technology, produce a curve with 2 peaks.^4^ These differences limit the interchangeability of different formulations and have led to substantial differences in definitions of bioequivalence between the regulatory bodies in different countries.^9^

Despite approval based on bioequivalence to the branded osmotic release oral system (OROS)‐methylphenidate ER, some generic long‐acting methylphenidate options have shown significant differences in duration and efficacy. For example, several long‐acting options showed reduced plasma methylphenidate concentrations late in the PK profile (approximately 8 hours after dosing) compared to OROS‐methylphenidate ER.^7^ A randomized, double‐blind, crossover trial in adults with ADHD comparing branded OROS‐methylphenidate with generic novo‐methylphenidate ER‐C demonstrated that patients were more satisfied with the efficacy and side effects of the branded option than the generic one.^10^ Similar concerns about differences in efficacy between certain generic long‐acting methylphenidate formulations and the branded reference have led to the proposed withdrawal of approval of some generic versions by the Food and Drug Administration (FDA),^9^ highlighting the need for high‐quality PK data to evaluate long‐acting methylphenidate options. In particular, examining not only total drug exposure but also drug exposure during specific periods after dosing (area under the concentration‐time curve from time 0 to 3 hours after dosing [AUC_0‐3 h_], AUC from 3 to 7 hours after dosing [AUC_3‐7 h_], and AUC from 7 to 12 hours after dosing [AUC_7‐12 h_], for instance) have proven useful, such that the FDA now requires bioequivalence within each of these time parameters as a prerequisite for approval.

A long‐acting formulation of methylphenidate, designated ODX‐methylphenidate ER, is approved by the FDA for the treatment of patients with ADHD (Figure S1).^11^ This formulation is available in 18‐, 27‐, 36‐, 45‐, 54‐, 63‐, and 72‐mg tablets, increments of 9 mg yielding in‐between doses of 45 and 63 mg not offered in most formulations. ODX‐methylphenidate ER employs a similar osmotic pressure‐controlled release technology as OROS‐methylphenidate ER to deliver methylphenidate at a steady rate over the day.^11^, ^12^ Both also include an immediate‐release drug overcoat to provide a faster onset of action of the drug. ODX‐methylphenidate ER has a bilayer core structure with a drug layer and a push layer, whereas OROS‐methylphenidate ER has a trilayer structure containing 2 drug layers and a push layer. In both cases, the aqueous environment of the gastrointestinal tract allows water to enter the semipermeable membrane surrounding the core structure, causing expansion of the osmotically active excipients in the push layer and release of methylphenidate from the drug layer(s) through a laser‐drilled orifice. Because the semipermeable membrane controls the rate at which water enters the tablet core, drug delivery is also controlled. The biologically inert components of the tablet are eliminated in the stool.

Herein, we describe 2 studies comparing the PK profile and bioequivalence of 2 doses of ODX‐methylphenidate ER to equivalent doses of OROS‐methylphenidate ER in healthy volunteers under fasting conditions.

## Methods

### Study Design and Drugs

Both studies were conducted at Worldwide Clinical Trials Early Phase Services, LLC (San Antonio, TX, USA) in accordance with the principles expressed in the Declaration of Helsinki. The study protocols were reviewed and approved by the institutional review board IntegReview IRB (Austin, TX, USA). All subjects provided written informed consent.

Both trials were single‐dose, open‐label, randomized, 4‐period, 2‐treatment replicate trials comparing ODX‐methylphenidate hydrochloride ER (Relexxii, Vertical Pharmaceuticals, LLC, which uses the proprietary Osmodex technology to deliver the drug at a controlled rate over the day) in 2 periods to OROS‐methylphenidate hydrochloride ER (Concerta, Janssen Pharmaceuticals, Inc.) in 2 periods in healthy adult volunteers under fasted conditions. The first trial compared a single 72‐mg tablet of ODX‐methylphenidate ER to two 36‐mg tablets of OROS‐methylphenidate hydrochloride ER. The second trial compared a single 54‐mg tablet of ODX‐methylphenidate ER to a single 54‐mg tablet of OROS‐methylphenidate ER. Each drug administration was separated by a washout period of at least 7 days.

In both trials, the study drug was administered after a 10‐hour overnight fast, and subjects fasted for an additional 4 hours following dose administration. Water was allowed ad libitum except for 1 hour before through 1 hour after dosing. Standard meals were provided at approximately 4 and 10 hours after administration and then at appropriate times thereafter.

Blood samples were drawn at predose (0 hour) and every 30 minutes until 10 hours postdose and then at 11, 12, 14, 16, 24, and 36 hours after study treatment. Subjects were closely monitored for safety parameters throughout the study.

### Study Subjects

Study subjects were healthy adults between the ages of 18 and 45 years at the time of dosing with a body mass index between 18 and 32 kg/m^2^ at a weight of at least 59 kg. Female subjects agreed to use a reliable form of birth control from screening until 14 days after study completion. At screening, the subjects’ heart rate was required to be between 40 and 100 bpm, systolic blood pressure 90‐145 mm Hg, and diastolic blood pressure 50‐95 mm Hg measured sitting after 3 minutes of rest. Out‐of‐range vital signs could be repeated once. Subjects were excluded if they had a history or presence of clinically significant cardiovascular, respiratory, pulmonary, hepatic, renal, hematologic, gastrointestinal, endocrine, immunologic, dermatologic, neurologic, oncologic, psychiatric disease, or any condition that, in the opinion of the investigator, would have jeopardized the safety of the subject or the validity of the study results. Those with a history or presence of seizures, raised intraocular pressure, glaucoma, mania, hypomania, marked anxiety, motor tics, or recent significant symptoms of asthma (<5 years) and those with a history of priapism, clinically significant allergies, or substance abuse were also excluded. Female subjects who were pregnant or lactating were excluded. Subjects were also excluded if they had used any prescription medication (except for hormonal contraceptive or hormonal replacement therapy) within 14 days, or over‐the‐counter medication, nutritional or dietary supplements, herbal preparations, or vitamins within 7 days of the first dose of study medication.

### Laboratory Assessments and Statistical Procedures

Although neither staff nor patients were blinded to study treatment at the clinical site, bioanalytical laboratory personnel were blinded to treatment until the initial analysis was completed.

Blood samples were collected in Vacutainer tubes containing dipotassium ethylenediaminetetraacetic acid, placed in an ice bath immediately after collection, and kept on ice throughout processing. Samples were centrifuged at 3000 rpm for 10 minutes at 4°C, and the resulting plasma was harvested and frozen within 60 minutes of collection. For liquid/liquid extraction, 200 µL of plasma sample was mixed with 20 µL methylphenidate‐D3 internal standard. Twenty microliters of sodium bicarbonate buffer was added to adjust the pH, and samples were extracted with 0.5 mL ethyl acetate.

For measurement of methylphenidate in plasma, samples for liquid chromatography‐mass spectrometry were injected onto an API 4000 (Sciex) with settings declustering potential = 30, collision energy = 25, and collision cell exit potential = 15, and equipped with a normal phase high‐performance liquid chromatography column. The peak area of the m/z 234‐84 methylphenidate product ion was measured against the peak area of the m/z 237‐84 methylphenidate‐D3 internal standard product ion. Quantitation was performed using a weighted linear least squares regression analysis generated from fortified plasma calibration standards prepared immediately before each run. The lower limit of quantification was 0.25 ng/mL of methylphenidate. Standard noncompartmental methods were used for PK analysis, and concentrations below the lower limit of quantification were set to 0. The within‐day coefficient of variation ranged from 1.3% to 6.4%, and bias ranged from −12.0% to −2.5%. Between‐day coefficient of variation ranged from 1.9% to 6.0%, and bias ranged from −9.3% to −4.0%.

Comparison of the natural log‐transformed PK parameters maximum drug concentration (C_max_), AUC_0‐3 h_, AUC_3‐7 h_, AUC_7‐12 h_, and AUC from time 0 extrapolated to infinity (AUC_inf_) for methylphenidate for ODX‐methylphenidate ER and OROS‐methylphenidate ER was done using either the 2 one‐sided tests procedure or the reference‐scaled procedure, depending on the within‐subject standard deviation of the reference product (s_WR_). Subjects completing both periods for the reference product were used to calculate s_WR._ Only subjects completing at least 2 study periods, 1 test and 1 reference, were included in the statistical analysis.

To determine the appropriate test for bioequivalence, the s_WR_ cutoff of 0.294 was used, corresponding to a within‐subject variability for the reference product of 30%.^13^ If the s_WR_ was less than 0.294, the 2 one‐sided tests procedure for a fully replicated design was used to determine bioequivalence for the individual PK parameters. Ninety percent confidence intervals (CIs) were constructed for the treatment ratios (ODX‐methylphenidate ER to OROS‐methylphenidate ER) of C_max_, AUC_0‐3 h_, AUC_3‐7 h_, AUC_7‐12 h_, and AUC_inf_. The point estimates and confidence limits were exponentiated back to the original scales, and bioequivalence was concluded if the CIs for the 3 parameters were contained within the limits of 0.8 and 1.25.

If s_WR_ was 0.294 or greater, the reference‐scaled procedure for a fully replicated design was used to determine bioequivalence for the individual PK parameters. Bioequivalence was concluded if both the following conditions were satisfied for C_max_, AUC_0‐3 h_, AUC_3‐7 h_, AUC_7‐12 h_, and AUC_inf_: (1) the 95% upper confidence bound for (Y_T_ − Y_R_)2 − θs^2^_WR_ was 0 or less, where Y_T_ is the natural log‐transformed AUC or C_max_ mean value for ODX‐methylphenidate and Y_R_ is the natural log‐transformed AUC or C_max_ value for OROS‐methylphenidate; and (2) the point estimate of the ODX‐methylphenidate/OROS‐methylphenidate mean ratio fell within the limits of 0.8 and 1.25.

## Results

For the 72‐mg study, 60 subjects were enrolled, and 55 completed all 4 study periods. Of the 5 subjects who discontinued, 2 withdrew consent due to adverse events (1 due to gastroenteritis and 1 due to diarrhea, discolored urine, and back pain), 1 was withdrawn due to protocol noncompliance (positive alcohol screen), and 2 withdrew for personal reasons.

For the 54‐mg study, 36 subjects were enrolled, and 32 completed all 4 study periods. Of the 4 subjects who discontinued, 2 withdrew consent due to personal reasons. Upon withdrawal, one of these patients disclosed having clinically significant asthma not reported at screening and, therefore, should have been excluded from the study. Two subjects were withdrawn due to protocol noncompliance (1 with a positive drug screen and 1 for a positive alcohol screen). The demographics of all subjects in both trials enrolled are shown in Table S1.

**Figure 1:**
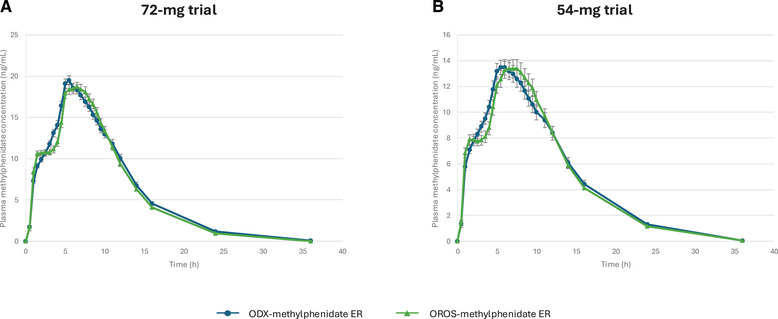
Mean plasma methylphenidate concentration over time following administration of ODX‐methylphenidate (blue) and OROS‐methylphenidate (green). Results from the 72‐mg trial are shown on the left, and those from the 54‐mg trial are shown on the right. Concentrations below the limit of quantification were set to 0. Error bars are standard error of the mean. Note that the 2 graphs have different y‐axis scales. ER, extended release; OROS, osmotic release oral system.

In the 72‐mg study, 56 subjects (93%) completed at least 2 study periods, including at least 1 dose of OROS‐methylphenidate ER, and were included in the PK analysis. Mean plasma methylphenidate concentration over time for ODX‐methylphenidate ER and OROS‐methylphenidate ER are shown in Figure 1A, and PK parameters are shown in Table 1. s_WR_ for the natural log‐transformed C_max_, AUC_0‐3 h_, AUC_3‐7 h_, AUC_7‐12 h_, and AUC_inf_ were 0.132, 0.149, 0. 102, 0.144, and 0.100, respectively, so the 2 one‐sided tests procedure was used (Table 2). The 90% CI for these parameters was within the prespecified range for establishing bioequivalence.

**Table 1:** PK Parameters for the 2 Trials Comparing Different ODX‐Methylphenidate ER Doses to Equivalent OROS‐Methylphenidate ER Doses

	72‐mg trial		54‐mg trial	
	ODX‐methylphenidate ER (n = 109)	OROS‐methylphenidate ER (n = 111)	ODX‐methylphenidate ER (n = 67)	OROS‐methylphenidate ER (n = 67)
T_max_ [median (range)], h	5.5 (3.5‐11.0)	6.5 (1.0‐10.0)	5.5 (2.5‐8.0)	7.0 (4.5‐10.0)
C_max_ (mean ± SD), ng/mL	20.6 ± 6.2	20.3 ± 7.4	14.5 ± 5.6	14.6 ± 6.0
AUC_0‐3 h_ (mean ± SD), ng•h/mL	22.4 ± 7.9	23.8 ± 8.1	17.4 ± 6.4	17.9 ± 6.6
AUC_3‐7 h_ (mean ± SD), ng•h/mL	67.0 ± 20.8	62.8 ± 21.3	48.1 ± 18.2	44.7 ± 18.1
AUC_7‐12 h_ mean ± SD, ng•h/mL	69.4 ± 26.3	71.7 ± 28.7	53.5 ± 25.1	57.2 ± 25.5
AUC_inf_ (mean ± SD), ng•h/mL	218.1 ± 83.8	210.9 ± 84.1	176.6 ± 82.4	173.8 ± 78.3
T_1/2_ (mean ± SD), h	4.0 ± 0.8	3.7 ± 0.7	4.5 ± 0.9	4.2 ± 0.8

These trials both had a 4‐period, 2‐treatment replicate design. In the 72‐mg trial, each subject was to receive one 72 mg tablet of ODX‐methylphenidate ER in 2 periods and two 36 mg tablets of OROS‐methylphenidate ER in 2 periods. In the 54 mg trial, each subject was to receive one 54‐mg tablet of ODX‐methylphenidate ER in 2 periods and one 54 mg tablet of OROS‐methylphenidate ER in 2 periods. Subjects were included in the PK analysis if they completed at least 2 study periods, including at least 1 dose of OROS‐methylphenidate ER. AUC_0‐3 h_, area under the concenetration‐time curve from time zero to 3 h after dosing; AUC_3‐7 h_, area under the concentration‐time curve from time 3 to 7 hours after dosing; AUC_7‐12 h_, area under the concentration‐time curve from 7 to 12 hours after dosing; AUC_inf_, area under the concentration‐time curve from time 0 extrapolated to infinity; C_max_, maximum concentration; ER, extended‐release; OROS, osmotic release oral system; SD, standard deviation; T_max_, time to maximum concentration; T_1/2_, elimination half‐life.

In the 54‐mg study, 34 subjects (94%) completed at least 2 study periods, including at least 1 dose of OROS‐methylphenidate ER, and were included in the PK analysis. Mean plasma methylphenidate concentration over time for ODX‐methylphenidate ER and OROS‐methylphenidate ER are shown in Figure 1B, and PK parameters are shown in Table 1. s_WR_ for the natural log‐transformed C_max_, AUC_0‐3 h_, AUC_3‐7 h_, AUC_7‐12 h_, and AUC_inf_ were 0.129, 0.139, 0. 123, 0.140, and 0.104, respectively, so the 2 one‐sided tests procedure was used (Table 2). The 90% CI for these parameters was within the prespecified range for establishing bioequivalence.

**Table 2:** Statistical Analysis of the Natural Log‐Transformed Systemic Exposure Parameters After Dosing With ODX‐Methylphenidate ER and OROS‐Methylphenidate ER

	72‐mg trial			54‐mg trial		
	Geometric mean ODX‐methylphenidate ER	Geometric mean OROS‐methylphenidate ER	Ratio ODX‐methylphenidate ER: OROS‐methylphenidate ER %, (90% CI)	Geometric mean ODX‐methylphenidate ER	Geometric mean OROS‐methylphenidate ER	Ratio ODX‐methylphenidate ER: OROS‐methylphenidate ER %, (90% CI)
ln(C_max_)	19.66	19.30	101.9 (98.9‐105.1)	13.76	13.77	99.9 (96.3‐103.7)
ln(AUC_3‐7 h_)	20.99	22.57	93.0 (90.1‐96.0)	16.48	16.86	97.7 (93.9‐101.8)
ln(AUC_3‐7 h_)	63.98	60.10	106.5 (103.9‐109.1)	45.60	42.07	108.4 (104.8‐112.1)
ln(AUC_7‐12 h_)	65.60	68.25	96.1 (93.2‐99.1)	49.55	53.53	92.6 (89.0‐96.3)
ln(AUC_inf_)	206.06	200.93	102.6 (100.5‐104.7)	162.87	161.39	100.9 (98.1‐103.8)

AUC_3‐7 h_, area under the concentration‐time curve from time 3 to 7 hours after dosing; AUC_7‐12 h_, area under the concentration‐time curve from 7 to 12 hours after dosing; AUC_inf_, area under the concentration‐time curve from time 0 extrapolated to infinity; C_max_, maximum concentration; CI, confidence interval; ER, extended‐release; ln, natural log‐transformed; OROS, osmotic release oral system.

In the 72‐mg trial, a total of 65 treatment‐emergent adverse events (TEAEs) were reported following dose administration in 21 subjects. All were considered mild or moderate in severity. The most common TEAEs were headache (7 events following dosage with ODX‐methylphenidate ER and 7 following dosage with OROS‐methylphenidate ER) and nausea (5 events following dosage with ODX‐methylphenidate ER and 3 following dosage with OROS‐methylphenidate ER). Two subjects were judged to have had TEAEs of clinical significance: One subject was diagnosed with neutropenia at the end‐of‐study clinical evaluations 36 hours after ODX‐methylphenidate ER dosing, and 1 subject experienced tachycardia during 3 of the study periods, twice following dosing with ODX‐methylphenidate ER and once after dosing with OROS‐methylphenidate ER. The TEAEs recorded in both studies are shown in Table S2.

In the 54‐mg trial, a total of 12 TEAEs were reported following dose administration in 10 subjects. Eleven were considered mild, and 1 was considered moderate in severity. Only one of these TEAEs was reported following dosing with ODX‐methylphenidate ER, with the remaining 11 reported following dosing with OROS‐methylphenidate ER. The most common TEAEs were headache (2 events, both following dosage with OROS‐methylphenidate ER) and viral syndrome (2 events, both following dosage with OROS‐methylphenidate ER). Two subjects were judged to have had TEAEs of clinical significance: 1 subject was diagnosed with neutropenia and elevated creatine kinase on early termination clinical laboratory evaluations performed approximately 10 days after OROS‐methylphenidate ER administration, and 1 subject experienced tachycardia approximately 4 hours after administration of ODX‐methylphenidate ER.

## Discussion

This PK and bioequivalence study was performed to evaluate a controlled‐release formulation of methylphenidate (ODX‐methylphenidate ER) compared to a previously available reference product (OROS‐methylphenidate ER). The 2 products are constructed similarly, with an immediate‐release drug layer to provide a rapid onset of drug action and an osmotic pressure system to deliver the drug at a controlled rate throughout the day. In these 4‐period replicate crossover studies in healthy adult subjects, ODX‐methylphenidate ER was found to be bioequivalent to OROS‐methylphenidate ER, both in terms of overall PK characteristics and drug exposure in each of the component intervals studied. All TEAEs recorded were mild or moderate in severity and were similar between the 2 drugs.

Despite differences in sex and race among the trial populations, the results of the 2 trials were very consistent. As expected, the overall drug exposure was reduced in the 54‐mg trial, and time to maximal plasma concentration of methylphenidate was similar. Overall rates of TEAEs were also largely similar between the trials.

Careful dose optimization of methylphenidate has been shown to provide significant symptom management benefits, especially in children, highlighting the need for ongoing medication management.^14^, ^15^ This management is made easier by the availability of multiple tablet strengths, such as those available for ODX‐methylphenidate ER. Given that the PK profile of methylphenidate has been shown to closely mirror its clinical effectiveness, small changes in PK parameters between formulations can have a significant effect on clinical utility.^7^ Some analyses have further shown that methylphenidate ER formulations that use osmotic pump technology for sustained drug delivery, as both the study drugs do in this trial, show better sustained symptom control than methylphenidate ER formulations that do not employ osmotic pump technology.^16^ PK profile differences and associated symptom management differences have been identified between different long‐acting formulations of methylphenidate,^17^ making switching between formulations potentially problematic for patients.

Various formulations of long‐acting methylphenidate have demonstrated similar drug exposure in the hours immediately following administration, likely due to the immediate‐release drug component included in each.^7^ Greater variability between formulations has been seen after 8 hours following dose administration, with some showing sustained efficacy whereas others do not.^7^ Many patients still require symptom control at this time of day, and decreased drug exposure during this period may necessitate an additional short‐acting dose late in the day, which can be cumbersome for patients. These studies showed bioequivalence between ODX‐methylphenidate ER and the reference OROS‐methylphenidate ER in AUC_7‐12 h_, suggesting these formulations should demonstrate similar late‐day clinical efficacy, which helped support the regulatory approval of ODX‐methylphenidate ER in the United States.

These studies have some limitations, namely, that they were conducted with healthy volunteers instead of participants with ADHD. Therefore, it was not possible to evaluate the correspondence between PK profile and therapeutic effect. In addition, these trials were performed under fasting conditions. Although this simplifies the comparison between the treatment arms, it does not reflect real‐world use, where patients often take their medication after eating.

In conclusion, these trials demonstrate that ODX‐methylphenidate ER is bioequivalent to the reference OROS‐methylphenidate ER, including in the critical 7‐12‐hours postdose window. The 2 treatments showed a similar safety and tolerability profile in healthy adult subjects.

## Conflicts of Interest

A.C.C. has received research support, served on an advisory board, as a consultant or speaker or has received writing support from the following: Aardvark, Acadia, Adlon, Akili, Allergan, Alora, Attentive, Axial, Cingulate, Corium, Emalex, Ironshore, Kempharm, Lumos, Neurocentria, Noven, Otsuka, Purdue, Receptor Life Sciences, Sunovion, Supernus, Takeda, Tris, and Tulex. A.A.‐S. declares a potential conflict of interest as an employee of Alora Pharmaceuticals. J.H.N. was a consultant/advisory board member for Adlon Therapeutics, Cingulate Therapeutics, Corium, Hippo T&C, Ironshore, Lumos, Medice, Mentavi Health, MindTension, Myriad, NLS, OnDosis, Otsuka, Rhodes, Shire/Takeda, Signant Health, and Supernus; he received research support from Cingulate, Ironshore, MindTension, Otsuka, and Supernus; honoraria for disease state lectures from Aspen Pharmaceuticals, Otsuka, and Shire, and served as a consultant for the US National Football League.

## Funding

This study was funded by Osmotica Kft. and was performed at the independent contract research organization Worldwide Clinical Trials Early Phase Services.

## Supporting information



Supporting Information

Supporting Information

## Data Availability

The data generated in this study are not publicly available due to their being confidential, proprietary company‐owned data but are available upon reasonable request from the corresponding author.

## References

[1] Faraone SV , Bellgrove MA , Brikell I , et al. Attention‐deficit/hyperactivity disorder. Nat Rev Dis Primers. 2024;10(1):11. 38388701 10.1038/s41572-024-00495-0

[2] Li Y , Yan X , Li Q , et al. Prevalence and trends in diagnosed ADHD among US children and adolescents, 2017–2022. JAMA Netw Open. 2023;6(10):E2336872. 37792379 10.1001/jamanetworkopen.2023.36872 PMC10551769

[3] Ayano G , Tsegay L , Gizachew Y , et al. Prevalence of attention deficit hyperactivity disorder in adults: umbrella review of evidence generated across the globe. Psychiatry Res. 2023;328:115449. 37708807 10.1016/j.psychres.2023.115449

[4] Childress AC , Komolova M , Sallee FR . An update on the pharmacokinetic considerations in the treatment of ADHD with long‐acting methylphenidate and amphetamine formulations. Expert Opin Drug Metab Toxicol. 2019;15(11):937‐974. 31581854 10.1080/17425255.2019.1675636

[5] Abbas K , Barnhardt EW , Nash PL , Streng M , Coury DL . A review of amphetamine extended release once‐daily options for the management of attention‐deficit hyperactivity disorder. Expert Rev Neurother. 2024;24(4):421‐432. 38391788 10.1080/14737175.2024.2321921

[6] Cortese S , Adamo N , Del Giovane C , et al. Comparative efficacy and tolerability of medications for attention‐deficit hyperactivity disorder in children, adolescents, and adults: a systematic review and network meta‐analysis. Lancet Psychiatry. 2018;5(9):727‐738. 30097390 10.1016/S2215-0366(18)30269-4 PMC6109107

[7] Coghill D , Banaschewski T , Zuddas A , Pelaz A , Gagliano A , Doepfner M . Long‐acting methylphenidate formulations in the treatment of attention‐deficit/hyperactivity disorder: a systematic review of head‐to‐head studies. BMC Psychiatry. 2013;13:237. 24074240 10.1186/1471-244X-13-237 PMC3852277

[8] O'Connor L , Carbone S , Gobbo A , Gamble H , Faraone SV . Pediatric attention deficit hyperactivity disorder (ADHD): 2022 updates on pharmacological management. Expert Rev Clin Pharmacol. 2023;16(9):799‐812. 37587841 10.1080/17512433.2023.2249414

[9] Moharram M , Kiang T . Pharmacokinetics of long‐acting methylphenidate: formulation differences, bioequivalence, interchangeability. Eur J Drug Metab Pharmacokinet. 2024;49(2):149‐170. 38127227 10.1007/s13318-023-00873-1

[10] Fallu A , Dabouz F , Furtado M , Anand L , Katzman MA . A randomized, double‐blind, cross‐over, phase IV trial of oros‐methylphenidate (CONCERTA^®^) and generic novo‐methylphenidate ER‐C (NOVO‐generic). Ther Adv Psychopharmacol. 2016;6(4):237‐251. 27536342 10.1177/2045125316643674 PMC4971598

[11] Patil P , Uphade K , Saudagar R . A review: osmotic drug delivery system. Pharma Science Monitor. 2018;9(2):283‐300.

[12] Conley R , Gupta SK , Sathyan G . Clinical spectrum of the osmotic‐controlled release oral delivery system (OROS), an advanced oral delivery form. Curr Med Res Opin. 2006;22(10):1879‐1892. 17022845 10.1185/030079906x132613

[13] Davit BM , Chen ML , Conner DP , et al. Implementation of a reference‐scaled average bioequivalence approach for highly variable generic drug products by the US Food and Drug Administration. AAPS J. 2012;14(4):915‐924. 22972221 10.1208/s12248-012-9406-x PMC3475857

[14] Vitiello B , Severe JB , Greenhill LL , et al. Methylphenidate dosage for children with ADHD over time under controlled conditions: lessons from the MTA. J Am Acad Child Adolesc Psychiatry. 2001;40(2):188‐196. 11211367 10.1097/00004583-200102000-00013

[15] Childress AC , Kollins SH , Cutler AJ , Marraffino A , Sikes CR . Open‐label dose optimization of methylphenidate extended‐release orally disintegrating tablet in a laboratory classroom study of children with attention‐deficit/hyperactivity disorder. J Child Adolesc Psychopharmacol. 2021;31(5):342‐349. 34081560 10.1089/cap.2020.0142

[16] Lally MD , Kral MC , Boan AD . Not all generic Concerta is created equal: comparison of OROS versus non‐OROS for the treatment of ADHD. Clin Pediatr (Phila). 2016;55(13):1197‐1201. 26467563 10.1177/0009922815611647

[17] Maldonado R . Comparison of the pharmacokinetics and clinical efficacy of new extended‐release formulations of methylphenidate. Expert Opin Drug Metab Toxicol. 2013;9(8):1001‐1014. 23611637 10.1517/17425255.2013.786041

