# Supplementary material for: Pharmacokinetics and Bioequivalence of a Novel Extended‐Release Formulation of Methylphenidate Hydrochloride for Attention‐Deficit/Hyperactivity Disorder
Source: Clin Pharmacol Drug Dev. 2025 Aug 12;14(11):829–35. doi: 10.1002/cpdd.1577 (PMC12583981; doi:10.1002/cpdd.1577)
Supplement: Supplementary file 1 — Supporting Information [file CPDD-14-829-s001.docx]

**Supplementary Digital Content for**

**Pharmacokinetics and Bioequivalence of a Novel Extended-Release Formulation of Methylphenidate Hydrochloride for Attention Deficit Hyperactivity Disorder**

Ann C Childress, Ahmad AL-Sabbagh, Jeffrey H Newcorn

# Supplementary Figure Legend

**Supplementary Figure 1: The ODX-methylphenidate ER drug delivery system.** When ingested, the immediate-release drug layer dissolves within 1 h. Subsequently, the semi-permeable membrane allows water to enter the tablet core at a controlled rate. This water absorption causes expansion of the osmotic push layer, resulting in a controlled rate of drug release through the laser-drilled orifice.

**Supplementary Table 1: Demographics of subjects enrolled in studies.**

|  | **72-mg trial N = 60** | **54-mg trial N = 36** |
| --- | --- | --- |
| **Age (mean ± SD, years)** | 31 ± 6 | 30 ± 6 |
| **Sex (%)** |  |  |
| Female | 56.7 | 27.8 |
| Male | 43.3 | 72.2 |
| **Race/Ethnicity (%)** |  |  |
| Hispanic/Latino-Black | 3.3 | 2.8 |
| Hispanic/Latino-White | 60.0 | 27.8 |
| American Indian/Alaska Native | 1.7 | 0 |
| Hispanic/Latino-American Indian/Alaska Native | 0 | 5.6 |
| Non-Hispanic/Latino-Black | 18.3 | 44.4 |
| Non-Hispanic/Latino-White | 15.0 | 19.4 |
| Non-Hispanic/Latino-White, Black | 1.7 | 0 |
| **BMI (mean ± SD, kg/m^2^)** | 27.6 ± 2.8 | 28.1 ± 2.6 |

SD, standard deviation.

**Supplementary Table 2: Treatment-emergent adverse events.**

|  | **72-mg trial** | | | | **54-mg trial** | | | |
| --- | --- | --- | --- | --- | --- | --- | --- | --- |
|  | **ODX-methylphenidate ER** **(n = 115)** | | **OROS-methylphenidate ER  (n = 113)** | | **ODX-methylphenidate ER** **(n = 69)** | | **OROS-methylphenidate ER (n = 67)** | |
|  | Number of subjects | Number of events | Number of subjects | Number of events | Number of subjects | Number of events | Number of subjects | Number of events |
| **Blood and lymphatic system disorders** | | | | | | | | |
| Neutropenia | 1 | 1 | 0 | 0 | 0 | 0 | 1 | 1 |
| **Cardiac disorders** | | | | | | | | |
| Heart palpitations | 0 | 0 | 1 | 1 | 0 | 0 | 0 | 0 |
| Palpitations | 0 | 0 | 1 | 1 | 0 | 0 | 0 | 0 |
| Tachycardia | 1 | 2 | 1 | 1 | 1 | 1 | 0 | 0 |
| **Gastrointestinal disorders** | | | | | | | | |
| Diarrhea | 1 | 1 | 0 | 0 | 0 | 0 | 0 | 0 |
| Dry mouth | 1 | 1 | 2 | 3 | 0 | 0 | 0 | 0 |
| Emesis | 1 | 2 | 1 | 2 | 0 | 0 | 0 | 0 |
| Gastroenteritis | 1 | 1 | 0 | 0 | 0 | 0 | 0 | 0 |
| Nausea | 4 | 5 | 2 | 3 | 0 | 0 | 0 | 0 |
| Toothache | 0 | 0 | 1 | 1 | 0 | 0 | 0 | 0 |
| Vomiting | 1 | 1 | 0 | 0 | 0 | 0 | 0 | 0 |
| **Infections and infestations** | | | | | | | | |
| Pharyngitis | 0 | 0 | 0 | 0 | 0 | 0 | 1 | 1 |
| Viral syndrome | 0 | 0 | 0 | 0 | 0 | 0 | 2 | 2 |
| **Investigations** | | | | | | | | |
| Elevated creatine kinase | 0 | 0 | 0 | 0 | 0 | 0 | 1 | 1 |
| **Metabolism and nutrition disorders** | | | | | | | | |
| Anorexia | 1 | 1 | 2 | 2 | 0 | 0 | 0 | 0 |
| **Musculoskeletal and connective tissue disorders** | | | | | | | | |
| Back pain | 1 | 1 | 0 | 0 | 0 | 0 | 0 | 0 |
| Left shoulder pain | 0 | 0 | 1 | 1 | 0 | 0 | 0 | 0 |
| Muscle spasm | 0 | 0 | 1 | 1 | 0 | 0 | 0 | 0 |
| Right hip pain | 0 | 0 | 1 | 1 | 0 | 0 | 0 | 0 |
| Weakness | 0 | 0 | 1 | 1 | 0 | 0 | 0 | 0 |
| **Nervous system disorders** | | | | | | | | |
| Dizziness | 1 | 1 | 1 | 2 | 0 | 0 | 0 | 0 |
| Drowsiness | 0 | 0 | 1 | 1 | 0 | 0 | 0 | 0 |
| Headache | 6 | 7 | 6 | 7 | 0 | 0 | 2 | 2 |
| Lightheaded | 0 | 0 | 0 | 0 | 0 | 0 | 1 | 1 |
| **Psychiatric disorders** | | | | | | | | |
| Agitation | 0 | 0 | 2 | 2 | 0 | 0 | 0 | 0 |
| Anxiety | 0 | 0 | 1 | 1 | 0 | 0 | 1 | 1 |
| Anxious | 1 | 1 | 1 | 1 | 0 | 0 | 0 | 0 |
| Euphoria | 1 | 1 | 0 | 0 | 0 | 0 | 1 | 1 |
| Insomnia | 0 | 0 | 1 | 1 | 0 | 0 | 0 | 0 |
| Restlessness | 0 | 0 | 1 | 1 | 0 | 0 | 0 | 0 |
| **Renal and urinary disorders** | | | | | | | | |
| Discolored urine | 1 | 1 | 0 | 0 | 0 | 0 | 0 | 0 |
| **Reproductive and breast disorders** | | | | | | | | |
| Irregular menses | 0 | 0 | 1 | 1 | 0 | 0 | 0 | 0 |
| **Respiratory, thoracic and mediastinal disorders** | | | | | | | | |
| Bronchitis | 0 | 0 | 1 | 1 | 0 | 0 | 0 | 0 |
| Nasal congestion | 0 | 0 | 0 | 0 | 0 | 0 | 1 | 1 |
| **Skin and subcutaneous tissue disorders** | | | | | | | | |
| Contact dermatitis | 1 | 1 | 0 | 0 | 0 | 0 | 0 | 0 |
| Pruritic rash | 0 | 0 | 1 | 1 | 0 | 0 | 0 | 0 |
