# Supplementary figures and images for: Pharmacokinetics and Bioequivalence of a Novel Extended‐Release Formulation of Methylphenidate Hydrochloride for Attention‐Deficit/Hyperactivity Disorder
Source: Clin Pharmacol Drug Dev. 2025 Aug 12;14(11):829–35. doi: 10.1002/cpdd.1577 (PMC12583981; doi:10.1002/cpdd.1577)

Supplementary Figure 1

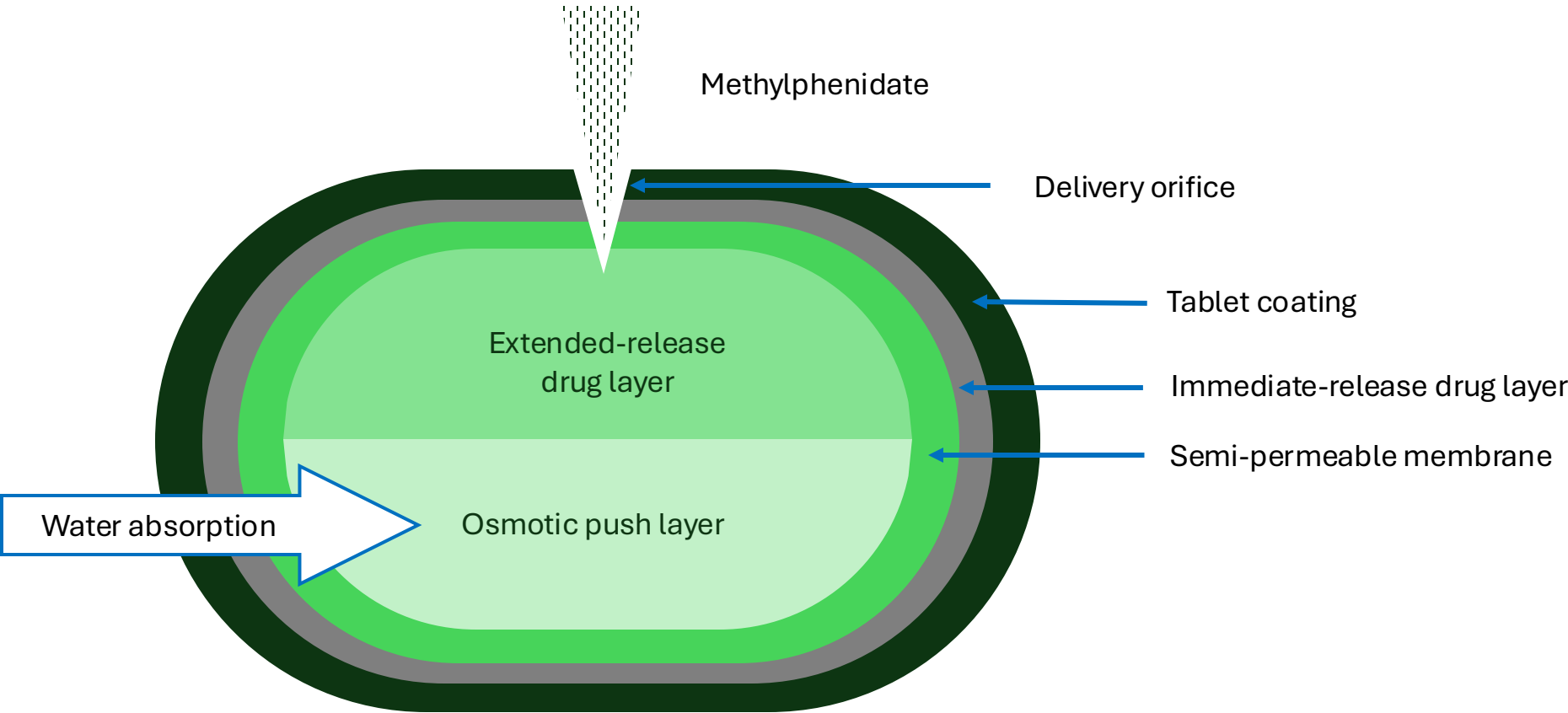

Supplement: Supplementary file 2 — Supporting Information [file CPDD-14-829-s002.pdf]
